# Beyond Bifid and Trifid: CBCT Findings of a Pentafid Mandibular Condyle in a Patient With a History of Childhood Trauma: A Case Report

**DOI:** 10.1155/crid/8674693

**Published:** 2026-08-26

**Authors:** Iremnur Sambur, Ismail Hakan Avsever, Hilal Peker Ozturk, Edanur Kaya

**Affiliations:** ^1^ Department of Dentomaxillofacial Radiology, Gulhane Faculty of Dentistry, University of Health Sciences, Ankara, Turkey, uhsr.ac.in

**Keywords:** case report, CBCT, condyle anomaly, mandibular condyle, multiheaded condyle

## Abstract

The mandibular condyle may exhibit a variety of morphological variations, most commonly bifid and, less frequently, trifid and tetrafid configurations. Although these anomalies are often detected incidentally and remain asymptomatic, some patients may present with temporomandibular joint dysfunction, pain, restricted mandibular movements, or facial asymmetry. We report the clinical and radiographic findings of a 50‐year‐old female patient in whom an unusual five‐lobed (pentafid) morphology of the right mandibular condyle was incidentally detected during routine radiographic examination. The patient had a history of childhood facial trauma and presented with mild temporomandibular joint symptoms, including limited mouth opening, pain during maximal mouth opening, occasional pain during mastication, and mandibular deviation during opening. Cone‐beam computed tomography (CBCT) confirmed the presence of five distinct condylar lobules, whereas the contralateral condyle demonstrated normal morphology. Owing to the absence of severe functional impairment, conservative management was recommended. To the best of our knowledge, no previously published case describing a pentafid mandibular condyle has been identified in the available literature. This case illustrates an unusual morphological manifestation within the spectrum of multiheaded mandibular condyles. Although the patient′s history suggests a possible association with childhood trauma, the absence of contemporaneous clinical and radiographic records precludes establishing a causal relationship.

## 1. Introduction

The temporomandibular joint (TMJ) plays a central role in mandibular function and is one of the most anatomically and functionally complex joints in the human body. The mandibular condyle, as a key component of this joint, can exhibit various morphological variations due to developmental, traumatic, or idiopathic factors [1]. Normal mandibular condyle development depends on coordinated endochondral ossification and remodeling during embryogenesis. Disturbances in these developmental processes, including persistence of embryonic fibrous septa or alterations in vascularization and ossification, have been proposed as possible mechanisms underlying multiheaded condylar morphology. In addition, posttraumatic remodeling following condylar injury has also been suggested as a potential etiological pathway for these rare anatomical variations [2–6]. In the literature, the term “multiheaded condyle” (MHC) is used to describe a relatively rare morphological anomaly in which the mandibular condyle is divided into multiple heads [2]. These variations typically present as bifid mandibular condyle (BMC) or trifid mandibular condyle (TMC) forms [7]. Although extremely rare, a few case reports have described the presence of tetrafid condyles [7–9]. Such variations are most often detected incidentally during routine radiographic examinations [1, 10–13]. However, due to the two‐dimensional limitations of panoramic radiographs, variations in condylar morphology may be overlooked. Therefore, three‐dimensional imaging modalities such as CBCT are essential for the detailed assessment of condylar morphology and the accurate diagnosis of rare anatomical variants [7, 10, 14].

A comprehensive literature search was conducted in PubMed, Scopus, Web of Science, and Google Scholar up to June 2026 using combinations of the keywords “bifid condyle,” “trifid condyle,” “tetrafid condyle,” “multiheaded mandibular condyle,” and “mandibular condyle variation.” To the best of our knowledge, no previously published case describing a five‐lobed (pentafid) mandibular condyle was identified. Therefore, the aim of this report was to present the clinical and radiographic characteristics of a pentafid mandibular condyle identified by CBCT and to discuss its possible etiological mechanisms and clinical implications in the context of the current literature.

## 2. Case Report

CBCT imaging was performed using a CARESTREAM CS 9600 unit (Carestream Health Inc., Rochester, New York, United States) under standard acquisition parameters (120 kVp, 5 mA, 24 s, dose‐area product: 1309 mGy cm^2^).

CBCT images were analyzed using CS Imaging Software (Carestream Health Inc., Rochester, New York, United States). Initially, three‐dimensional volume‐rendered reconstructions were evaluated to assess the overall three‐dimensional morphology of the mandibular condyle. Subsequently, multiplanar reconstruction (MPR) images, including axial, coronal, and sagittal sections, were examined in detail. Image interpretation was performed independently by four experienced oral and maxillofacial radiologists. The final diagnosis of a pentafid mandibular condyle was established by consensus after confirming the presence of five distinct condylar lobules on both the three‐dimensional reconstructions and multiplanar CBCT images. Each condylar lobule was considered a separate component when it demonstrated a distinct cortical outline and was separated from the adjacent lobules by clearly identifiable grooves.

**Figure 1:**
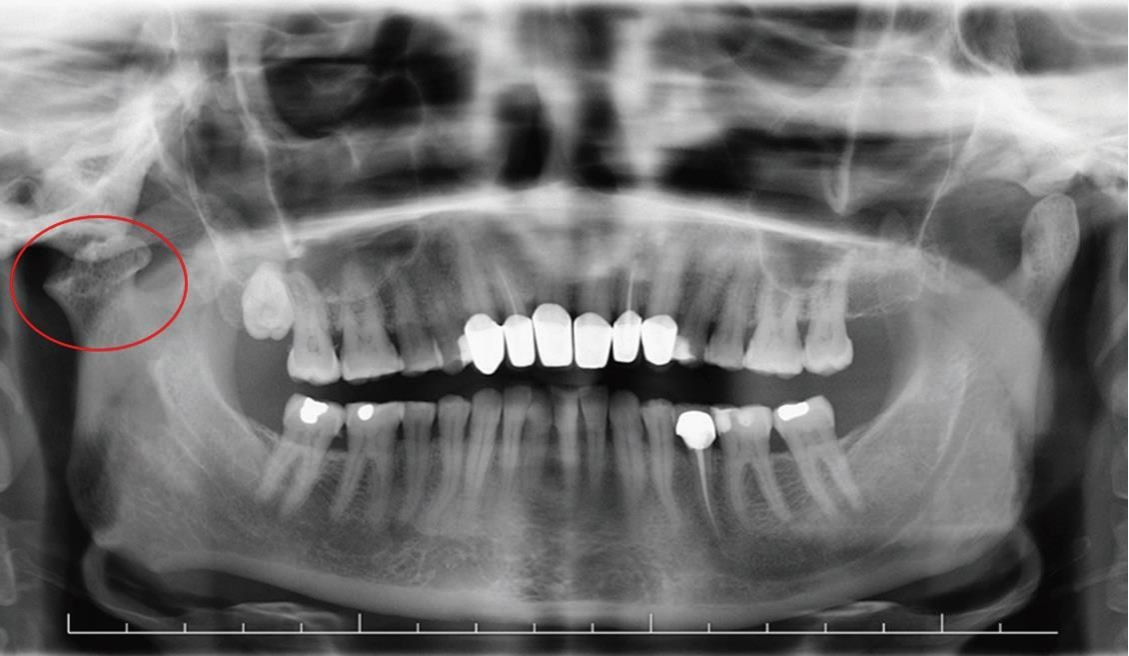
Panoramic radiograph demonstrating the incidental finding of a multilobulated right mandibular condyle.

**Figure 2:**
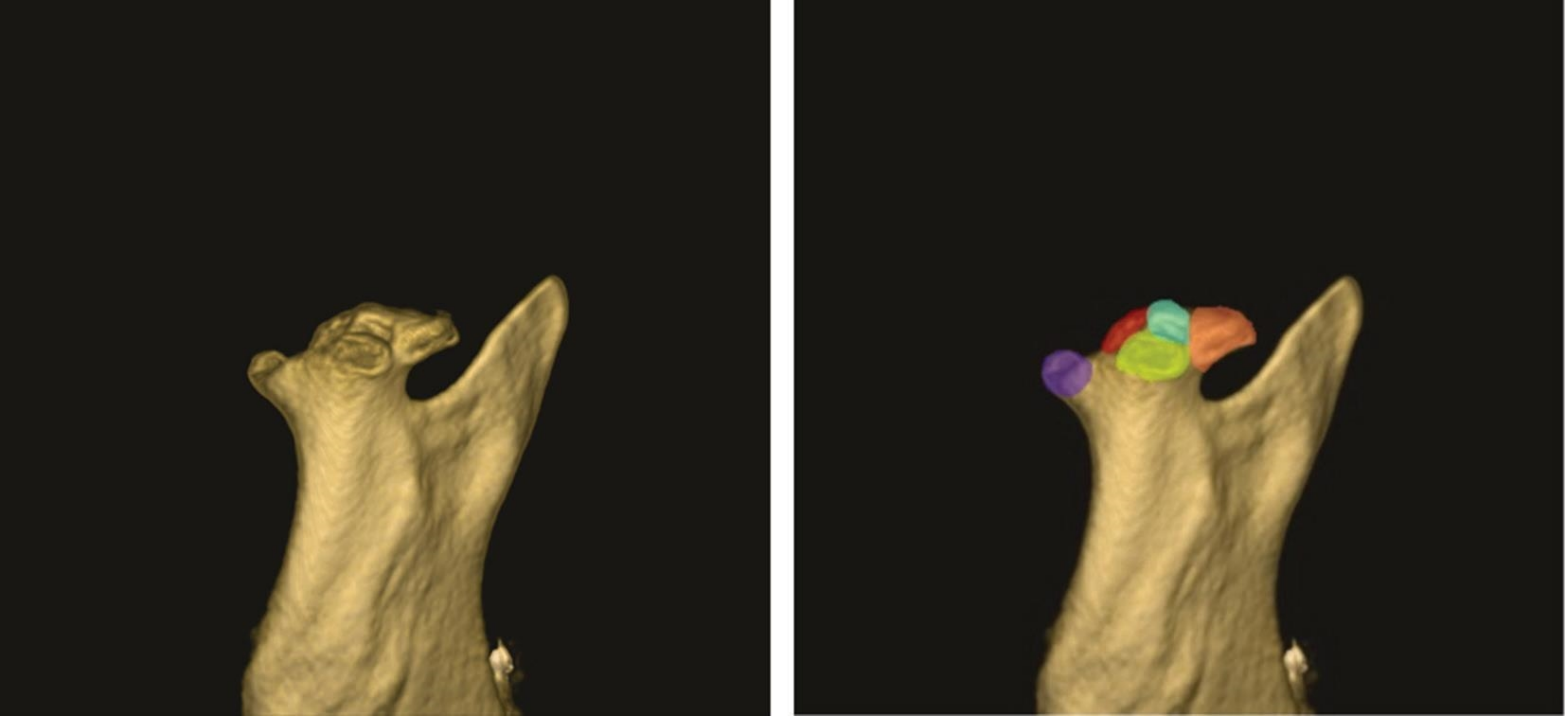
Three‐dimensional CBCT reconstruction of the right mandibular condyle alongside a color‐coded three‐dimensional rendering illustrating the five distinct condylar lobules.

**Figure 3:**
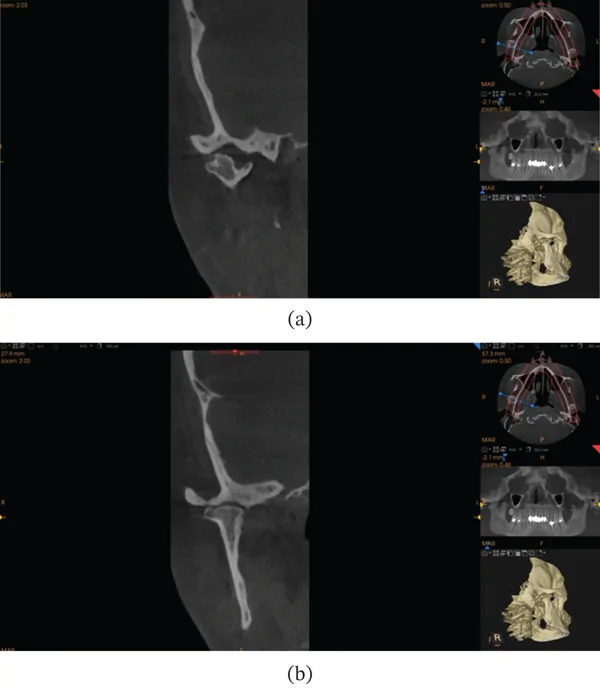
Sequential sagittal CBCT sections of the right mandibular condyle demonstrating the pentafid morphology. (a) Three distinct condylar heads are visible in the initial sagittal section, and (b) two additional heads appear in subsequent sagittal sections, confirming the presence of a total of five separate condylar heads.

**Figure 4:**
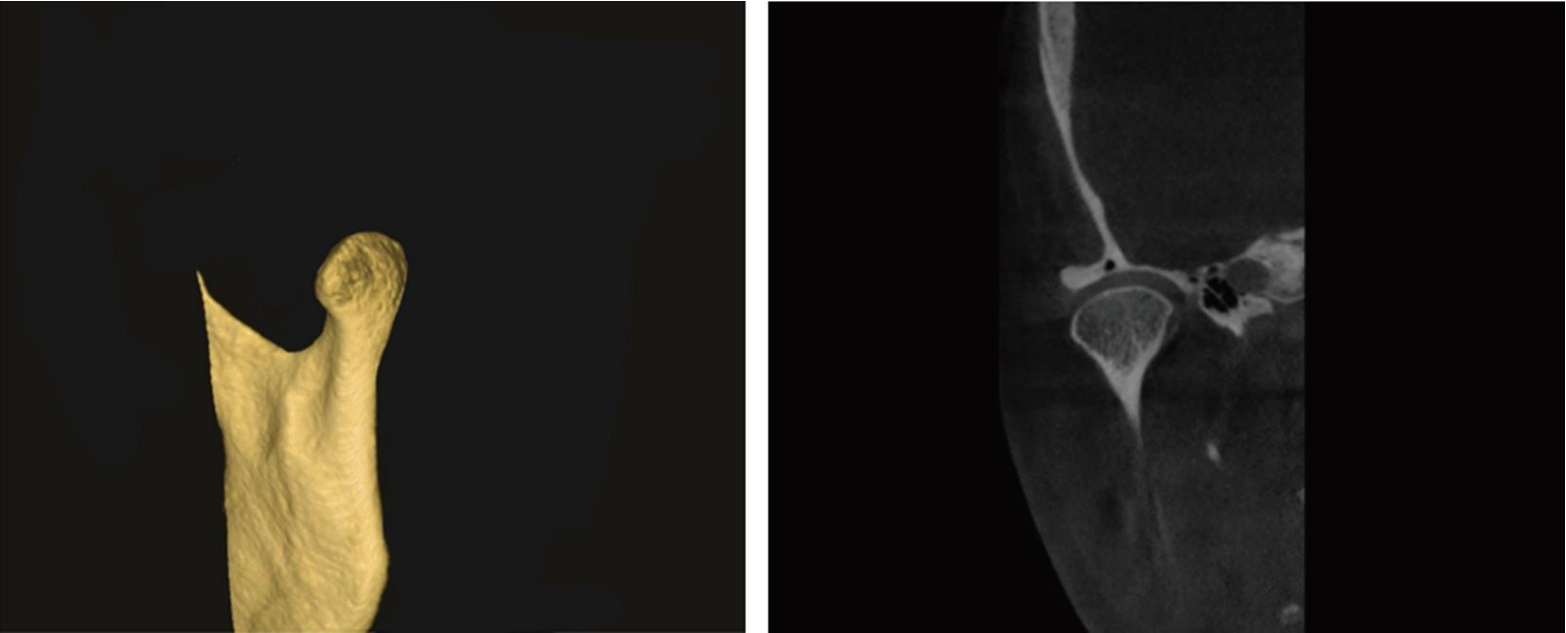
CBCT image of the left mandibular condyle showing normal single‐headed condylar morphology.

A 50‐year‐old female patient presented to our clinic for a routine dental examination. A panoramic radiograph incidentally revealed an unusual multiheaded morphology of the right mandibular condyle (Figure 1). Subsequent CBCT evaluation confirmed the presence of a pentafid right mandibular condyle. Three‐dimensional reconstructions clearly demonstrated the five distinct condylar lobules, allowing detailed assessment of the unusual morphology (Figure 2). CBCT demonstrated five distinct condylar lobules of varying sizes, separated by shallow grooves, without evidence of cortical disruption or other pathological osseous changes (Figure 3). The right condyle maintained its normal anatomical relationship with the glenoid fossa, with preservation of the joint space and no evidence of osseous fusion or ankylosis. The left condyle and TMJ also appeared within normal anatomical limits (Figure 4).

**Figure 5:**
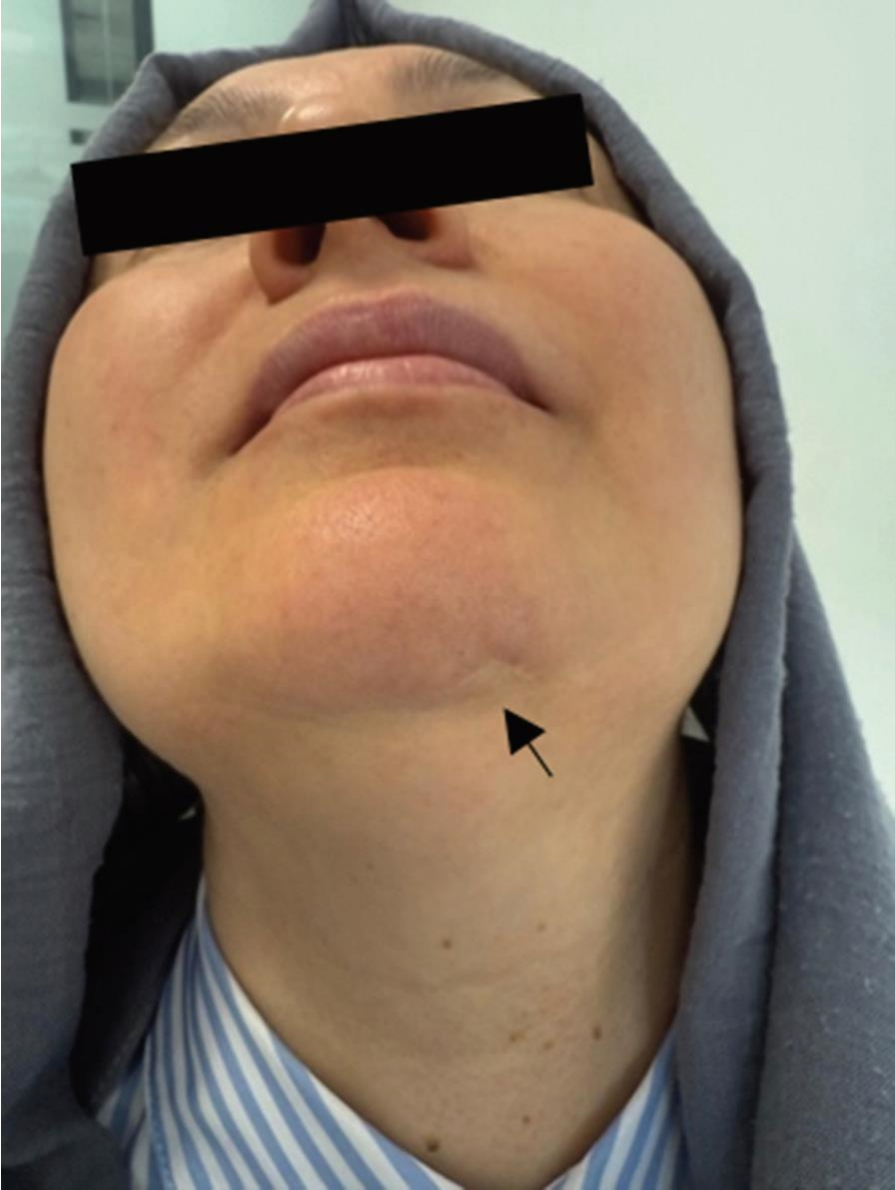
Scar on the chin of the patient resulting from trauma.

The patient′s medical history revealed a traumatic fall at approximately 4 years of age, resulting in bleeding from the chin and a residual scar on the left side of the chin (Figure 5). According to the patient′s recollection, no medical evaluation was sought following the injury. The bleeding was controlled at home, no radiographic examination was performed, and no mandibular fracture was diagnosed. The patient also reported that she had been unaware of the unusual condylar morphology and had never previously been informed of this anatomical variation. According to the patient′s knowledge, no family members had a history of similar mandibular condylar morphology, TMJ disorders, or congenital craniofacial anomalies.

**Figure 6:**
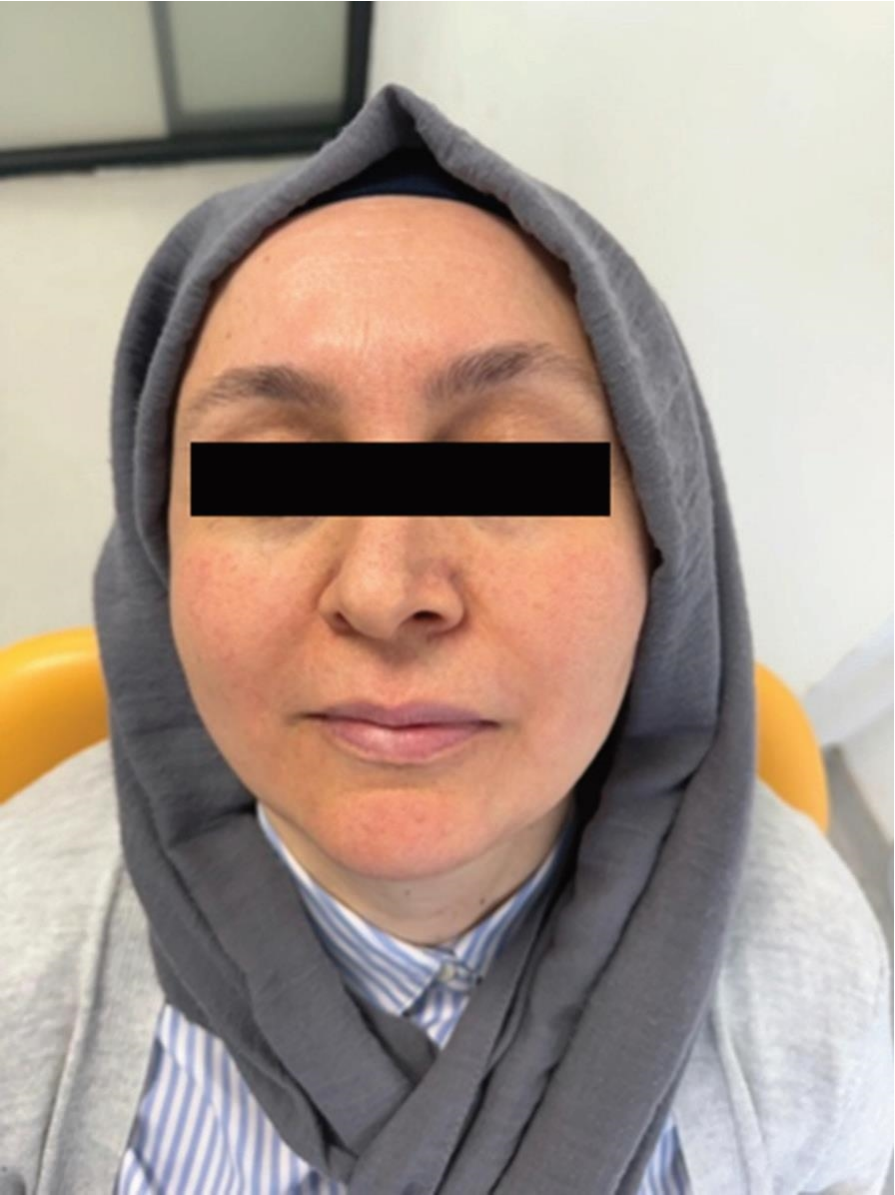
Extraoral view of the patient with the mouth closed.

**Figure 7:**
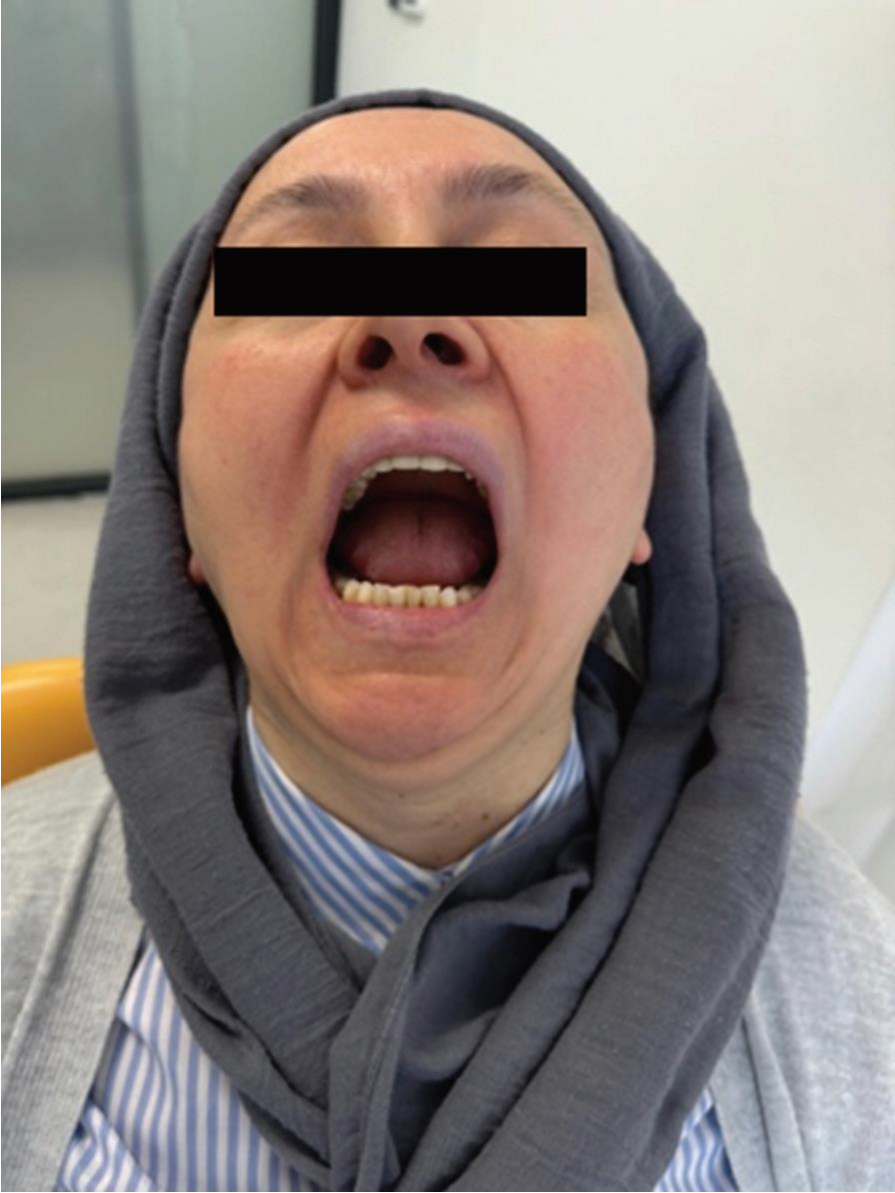
Extraoral view of the patient with the mouth open, demonstrating deviation to the right.

**Figure 8:**
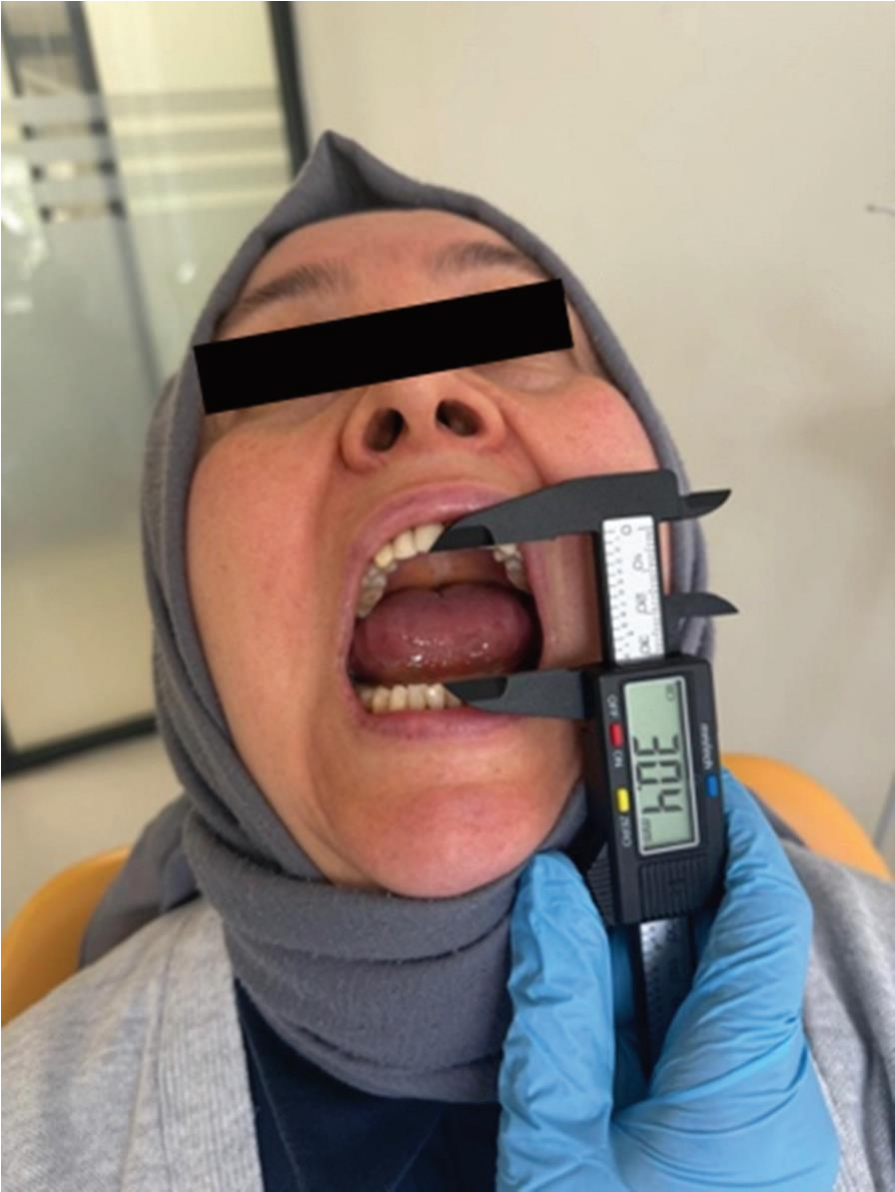
Clinical photograph demonstrating the patient′s maximum mouth opening.

Clinical examination revealed no apparent facial asymmetry with the mouth closed (Figure 6). During mouth opening, mild deviation of the mandible toward the right side was observed (Figure 7). The maximum unassisted mouth opening, measured using a digital caliper, was 30.4 mm (Figure 8). Right lateral excursion was clinically within normal limits, whereas left lateral excursion was restricted. During protrusive movement, the mandible deviated toward the right side. The patient reported occasional joint sounds during mandibular movements; however, no clicking or crepitus was detected during the clinical examination. Palpation of both TMJs did not elicit tenderness. Although the patient reported no spontaneous TMJ pain during daily activities, pain was elicited in the right TMJ during maximal mouth opening. On further questioning, she acknowledged occasional pain during mastication and bruxism‐related symptoms; however, she stated that these symptoms had not significantly affected her daily activities.

Considering the absence of severe functional impairment or significant pathological findings, conservative management was recommended. The patient was informed about the radiological findings and advised to use an occlusal splint and adopt behavioral modifications to reduce bruxism‐related parafunctional habits. At the follow‐up visit, the patient reported that she had not used the recommended occlusal splint because her symptoms were mild and did not significantly affect her daily activities. She was counseled again regarding the potential benefits of occlusal splint therapy and advised to continue clinical follow‐up. At the most recent follow‐up, her symptoms remained stable, and no deterioration in mandibular function was observed (Table 1).

**Table 1:** Clinical timeline.

Time	Clinical event
Approximately 4 years of age	Facial trauma resulting in bleeding from the chin; no medical evaluation or radiographic examination was performed.
Childhood–adulthood	The patient remained unaware of the condylar morphological variation and reported no significant temporomandibular complaints.
Age 50 years	Routine panoramic radiograph incidentally revealed an unusual morphology of the right mandibular condyle.
Same visit	CBCT examination confirmed a five‐lobed (pentafid) mandibular condyle.
Clinical assessment	Maximum mouth opening of 30.4 mm, right TMJ pain during maximal opening, restricted left lateral excursion, and mandibular deviation during opening were recorded.
Management	Conservative treatment was recommended, including an occlusal splint and behavioral modifications.
Follow‐up	The patient reported that she had not used the occlusal splint, and her symptoms remained stable.

## 3. Discussion

Bifid condyle was first described by Hrdlicka as a mandibular condyle divided into two parts by a sulcus of varying depth. This division may present as a shallow groove or, in some cases, as a distinct cleft [2]. Blackwood examined the developing condylar cartilage in human fetuses and identified well‐vascularized fibrous septa oriented anteroposteriorly within the condyle, which typically regress around the 19th week of gestation. He proposed that these septa normally demarcate the condylar head during development, and that if ossification fails or vascular compromise occurs, bifidity of the condyle may ensue [2, 3]. In an experimental condylectomy study on monkeys, Poswillo [4] suggested that alterations in the position of the articular disc could lead to the formation of interarticular septa within the condylar space, potentially triggering the development of a new condyle. This study demonstrated that posttraumatic healing and remodeling of condylar fractures may result in bifid‐like condylar appearances [7]. Later, Szentpetery et al. [5] proposed that in cases where the condyle is divided parallel to the coronal plane, failure of ossification of a developmental fibrous septum may be implicated, whereas in divisions parallel to the sagittal plane, trauma may play a primary etiologic role in the formation of bifid condyle.

Although the exact etiology of condylar morphological variations remains uncertain, several contributing factors have been proposed, including trauma, teratogenic agents, nutritional deficiencies, genetic predisposition, endocrine disorders, infections, and radiation exposure [1, 3, 7, 9, 10, 13, 15, 16]. Among these, trauma is the most consistently supported etiological factor in the current literature [2, 10, 17]. This hypothesis is reinforced by numerous case reports. For instance, Stadnicki [18] reported a case of a 3‐year‐old girl with unilateral BMC and TMJ ankylosis, attributed to a forceps‐assisted delivery. This remains the only documented case to directly associate bifid condyle formation with forceps trauma. In contrast, most other cases report a history of facial or mandibular trauma during early childhood rather than perinatal causes. Ertas et al. [19] described bilateral bifid condyles in a 5‐year‐old girl who had fallen from a height at the age of two. Similarly, Zoabi et al. [20] reported a case of unilateral trifid condyle in a 26‐year‐old woman with a history of facial trauma secondary to a fall at the age of 4.

Although trauma is widely considered the most common etiologic factor, several case series and radiographic screening studies have reported no history of trauma in the majority of patients [12]. For example, Miloglu et al. [11] evaluated panoramic radiographs of 10,200 individuals and identified BMC in 32 cases (0.3%), none of whom reported a history of trauma. Similarly, Menezes et al. [21] reviewed 50,080 panoramic radiographs and detected BMC in only 9 cases (0.018%), again with no reported trauma. One of the most compelling pieces of evidence supporting nontraumatic etiologic hypotheses comes from an experimental study by Gundlach et al. [6], in which the administration of teratogenic agents—such as N‐methyl‐N‐nitrosourea and formhydroxamic acid—at various stages of gestation induced the formation of bifid condyles in animal models. This study provides strong experimental support for the hypothesis that teratogenic factors may disrupt normal condylar morphogenesis [12]. In addition to trauma‐related cases, rare nontraumatic associations with BMC have also been documented. For instance, Motta‐Junior et al. [22] reported the first known case of bilateral trifid condyles in a 17‐year‐old male patient with congenital Frey syndrome and no history of trauma. Taken together, these observations suggest that trauma alone cannot explain every case of multiheaded condylar morphology and that developmental or other multifactorial mechanisms should also be considered.

The reported prevalence of BMC in the literature varies widely, depending on the imaging modality employed, the demographic characteristics of the studied population, and differences in sample size. The lowest prevalence to date was reported by Menezes et al. [21], who identified a rate of 0.018% through the evaluation of panoramic radiographs. In contrast, Kempwade et al. [1] also used panoramic imaging and reported the highest prevalence in the literature, at 6%. Studies utilizing CBCT have demonstrated intermediate prevalence rates; for example, Sampaio‐Neves et al. [23] reported a prevalence of 1.1%, whereas Khojastepour et al. [24] found a prevalence of 4.53%. These findings represent the lowest and highest BMC prevalence rates documented using panoramic radiography and CBCT, respectively. The mandibular condyle is a clinically significant anatomical structure due to its morphological variations and potential pathological conditions. However, because panoramic radiography is commonly used in routine dental examinations, certain anomalies involving the condylar region may be overlooked due to the inherent limitations of two‐dimensional imaging. Factors such as superimposition and distortion can hinder the accurate detection of morphological variations like BMC. Therefore, advanced imaging modalities are essential for the precise diagnosis of BMC and detailed evaluation of its structural characteristics. An increasing number of studies in the literature have emphasized the role of CT, and particularly CBCT, in the assessment of BMC. Owing to its three‐dimensional imaging capabilities, CBCT allows for more accurate, detailed, and reliable analysis of condylar morphology [7, 16, 24].

In clinical practice, suspected cases of BMC are often initially identified on panoramic radiographs, with CBCT subsequently used for detailed assessment and definitive diagnosis [14, 16]. With the increasing availability and use of advanced imaging technologies such as CBCT, the detection rate of bifid and trifid condyles has risen markedly. Although earlier reports documented approximately 110 cases of bifid condyles and only four cases of trifid condyles [9], more recent studies have identified as many as 270 bifid and 14 trifid condyles, reflecting this upward trend. In addition, three cases of tetrafid condyles have also been reported in the literature, according to a PubMed database review.

The presence of symptoms in patients with BMC has been reported with considerable variability across the literature. Although Loh and Yeo [12] observed that approximately 67% of cases were asymptomatic, Borrás‐Ferreres et al. [3], in their analysis of 216 BMC cases, reported clinical symptoms in 59.4% of patients. The most commonly observed findings included hypomobility (22.7%), arthralgia (18.1%), joint sounds (17.2%), and ankylosis (17.6%). Additional case reports have documented a wide spectrum of clinical manifestations, including joint noises, pain, restricted mandibular motion, trismus, swelling, ankylosis, and facial asymmetry [10, 15]. In symptomatic cases, treatment should be tailored to the patient′s specific complaints. When joint stability is maintained, conservative approaches such as occlusal splint therapy and arthroscopy may be effective. However, in the presence of ankylosis, surgical intervention—including condylectomy or arthroplasty—may be required [3, 10, 25].

Previously reported trifid and tetrafid mandibular condyles share several similarities with the present case. Most have been detected incidentally, with patients presenting either no symptoms or only mild temporomandibular complaints. Conservative management has generally been sufficient in the absence of ankylosis or severe functional impairment. Similar to previously reported trifid and tetrafid condyles, our patient presented with only mild clinical findings despite the markedly unusual condylar morphology. Although the present case showed a greater number of condylar lobules than previously reported trifid and tetrafid condyles, its clinical presentation remained relatively mild. The cortical contours were preserved, articulation with the glenoid fossa appeared normal, and no evidence of ankylosis or significant functional impairment was observed. This suggests that increasing morphological complexity is not necessarily accompanied by greater clinical severity.

A review of the available literature did not identify any previously published report describing a mandibular condyle composed of five distinct lobules. Rather than representing a new pathological entity, the term pentafid is used here only to describe the observed anatomical configuration. The present finding may therefore represent an uncommon morphological manifestation within the spectrum of multiheaded mandibular condyles. Because only a single case is currently available, additional reports will be required to better define the morphological spectrum and biological significance of this finding.

Compared with previously reported trifid and tetrafid condyles, the present case demonstrated five distinct condylar lobules while maintaining preserved cortical integrity, normal articulation with the glenoid fossa, and minimal functional impairment. These findings further support the concept that increasing morphological complexity does not necessarily correlate with greater clinical severity.

Considering the patient′s history of significant childhood trauma, the present morphology may be more appropriately interpreted as the structural outcome of adaptive posttraumatic remodeling rather than as a distinct developmental anatomical variant. From this perspective, the presence of five condylar lobules likely represents an extreme morphological expression within the same biological reparative continuum previously described in bifid, trifid, and tetrafid condyles, rather than a distinct developmental subtype. Although the number of condylar lobules is greater than in previously reported cases, the present patient exhibited a similar clinical course, conservative management, and favorable functional status. Therefore, the available evidence does not suggest that increasing the number of condylar lobules represents a separate pathological entity or confers distinct clinical or prognostic implications.

The patient′s history of childhood trauma supports the hypothesis that the observed morphology may represent extensive adaptive remodeling following condylar injury. Nevertheless, owing to the absence of contemporaneous imaging or medical documentation, this interpretation remains speculative, and a causal relationship cannot be established. Furthermore, because similar MHCs have also been reported in patients without a history of trauma, developmental or multifactorial mechanisms cannot be excluded.

## 4. Conclusion

This report describes an exceptionally rare pentafid mandibular condyle, which may represent an uncommon morphological manifestation of posttraumatic condylar remodeling within the spectrum of multiheaded mandibular condyles rather than a separate anatomical entity. To the best of our knowledge, no comparable case has been reported in the available literature. Although the patient′s history suggests a possible association with childhood trauma, the absence of contemporaneous clinical and radiographic records precludes establishing a causal relationship. This case highlights the value of comprehensive clinical assessment together with CBCT in the evaluation of unusual condylar morphology and emphasizes the need for additional well‐documented cases to improve understanding of the etiology, morphological spectrum, and clinical significance of these rare anatomical variations.

### 4.1. Patient Perspective

The patient stated that she had been unaware of the condylar variation before the present examination and that the associated symptoms had not significantly affected her daily life. For this reason, she was initially reluctant to use the recommended occlusal splint. Following a detailed explanation of the diagnosis, its possible etiology, and the rationale for conservative management, she understood the proposed treatment approach and agreed to continue with periodic clinical follow‐up.

### 4.2. Limitations

Several limitations should be considered when interpreting this case. First, as this report describes a single patient, the findings cannot be generalized to the broader population. Second, no historical radiographic examinations or medical records documenting the reported childhood trauma were available. Therefore, although the patient′s history suggests a possible association between the trauma and the observed condylar morphology, a causal relationship cannot be established. The retrospective nature of the trauma history also introduces the possibility of recall bias. Furthermore, the absence of longitudinal imaging precludes assessment of the temporal evolution of the condylar morphology. Finally, because the diagnosis was established exclusively on clinical and radiological findings, histopathological confirmation was neither available nor ethically justified. Additional well‐documented cases with long‐term clinical and radiographic follow‐up are required to further clarify the etiology, morphological spectrum, and clinical significance of this rare condylar variation.

## Author Contributions

**Iremnur Sambur:** conceptualization, methodology, software, investigation, data curation, writing – original draft, and visualization. **Ismail Hakan Avsever:** conceptualization, methodology, validation, writing – review and editing, and supervision. **Hilal Peker Ozturk:** validation and writing – review and editing. **Edanur Kaya:** software, writing – original draft, and writing – review and editing.

## Funding

No funding was received for this manuscript.

## Disclosure

All authors have read and approved the final version of the manuscript. Dr. İremnur Sambur, as the corresponding author, had full access to all of the data in this study and takes complete responsibility for the integrity of the data and the accuracy of the data analysis.

## Ethics Statement

This study was conducted in accordance with the principles of the Declaration of Helsinki. Ethical approval for publication of this case report was obtained from the Scientific Research Ethics Committee of the University of Health Sciences, Gulhane (Approval No. 2025‐521). Written informed consent was obtained from the patient for publication of the clinical information and radiographic images included in this report.

## Consent

Written informed consent was obtained from the patient for publication of this case report and the accompanying images.

## Conflicts of Interest

The authors declare no conflicts of interest.

## Data Availability

The data that support the findings of this study are available on request from the corresponding author. The data are not publicly available due to privacy or ethical restrictions.

## References

[1] Kempwade P. K. , Kattimani S. , Ramesh D. N. S. V. , Byatnal A. , Bansode A. , and Mujawar K. K. , Evaluation of the Prevalence of Bifid Mandibular Condyle Detected on Digital Panoramic Radiographs in North Karnataka Region, Journal of Advanced Clinical & Research Insights. (2019) 6, no. 5, 149–152, 10.15713/ins.jcri.281.

[2] Guven O. , A Study on Etiopathogenesis and Clinical Features of Multiheaded (Bifid and Trifid) Mandibular Condyles and Review of the Literature, Journal of Cranio-Maxillofacial Surgery. (2018) 46, no. 5, 773–778, 10.1016/j.jcms.2018.02.011, 29627366. 29627366

[3] Borrás-Ferreres J. , Sánchez-Torres A. , and Gay-Escoda C. , Bifid Mandibular Condyles: A Systematic Review, Medicina Oral, Patología Oral y Cirugía Bucal. (2018) 23, no. 6, e672–e680, 10.4317/medoral.22681, 30341271. 30341271 PMC6261007

[4] Poswillo D. E. , The Late Effects of Mandibular Condylectomy, Oral Surgery, Oral Medicine, Oral Pathology. (1972) 33, no. 4, 500–512, 10.1016/0030-4220(72)90361-1, 4622847. 4622847

[5] Szentpetery A. , Kocsis G. , and Marcsik A. , The Problem of the Bifid Mandibular Condyle, Journal of Oral and Maxillofacial Surgery. (1990) 48, no. 12, 1254–1257, 10.1016/0278-2391(90)90477-J, 2231143. 2231143

[6] Gundlach K. K. , Fuhrmann A. , and Beckmann-Van der Ven G. , The Double-Headed Mandibular Condyle, Oral Surgery, Oral Medicine, Oral Pathology. (1987) 64, no. 2, 249–251, 10.1016/0030-4220(87)90098-3, 3476903. 3476903

[7] Yelken K. M. , Goksel S. , and Ozcan I. , Multiheaded Mandibular Condyles, Journal of Orofacial Orthopedics. (2023) 84, no. Supplement 3, 165–171, 10.1007/s00056-022-00416-4, 35881143. 35881143

[8] Turkmenoglu A. and Yildirim B. , Tetrafid and Trifid Mandibular Condyle: A Case Report, Oral and Maxillofacial Surgery. (2025) 126, no. 4, 102191, 10.1016/j.jormas.2024.102191. 39644971

[9] Sahman H. , Etoz O. A. , Sekerci A. E. , Etoz M. , and Sisman Y. , Tetrafid Mandibular Condyle: A Unique Case Report and Review of the Literature, Dentomaxillofacial Radiology. (2011) 40, no. 8, 524–530, 10.1259/dmfr/62082661, 22065803. 22065803 PMC3528151

[10] Antoniades K. , Hadjipetrou L. , Antoniades V. , and Paraskevopoulos K. , Bilateral Bifid Mandibular Condyle, Oral Surgery, Oral Medicine, Oral Pathology, Oral Radiology, and Endodontics. (2004) 97, no. 4, 535–538, 10.1016/j.tripleo.2003.09.003. 15088041

[11] Miloglu O. , Yalcin E. , Buyukkurt M. , Yilmaz A. , and Harorli A. , The Frequency of Bifid Mandibular Condyle in a Turkish Patient Population, Dentomaxillofacial Radiology. (2010) 39, no. 1, 42–46, 10.1259/dmfr/38196548, 20089743. 20089743 PMC3520408

[12] Loh F. C. and Yeo J. F. , Bifid Mandibular Condyle, Oral Surgery, Oral Medicine, Oral Pathology. (1990) 69, no. 1, 24–27, 10.1016/0030-4220(90)90263-R. 2404225

[13] Sales M. A. , Oliveira J. X. , and Cavalcanti M. G. , Computed Tomography Imaging Findings of Simultaneous Bifid Mandibular Condyle and Temporomandibular Joint Ankylosis: Case Report, Brazilian Dental Journal. (2007) 18, no. 1, 74–77, 10.1590/S0103-64402007000100016, 17639206. 17639206

[14] Sahman H. , Sisman Y. , Sekerci A. E. , Tarim-Ertas E. , Tokmak T. , and Tuna I. S. , Detection of Bifid Mandibular Condyle Using Computed Tomography, Medicina Oral, Patología Oral y Cirugía Bucal.(2012) 17, no. 6, e930–e934, 10.4317/medoral.17748, 22549674. 22549674 PMC3505712

[15] Quayle A. A. and Adams J. E. , Supplemental Mandibular Condyle, British Journal of Oral and Maxillofacial Surgery. (1986) 24, no. 5, 349–356, 10.1016/0266-4356(86)90020-3, 2945583. 2945583

[16] Sahman H. , Sekerci A. E. , Ertas E. T. , Etoz M. , and Sisman Y. , Prevalence of Bifid Mandibular Condyle in a Turkish Population, Journal of Oral Science. (2011) 53, no. 4, 433–437, 10.2334/josnusd.53.433, 22167027. 22167027

[17] Artvinli L. B. and Kansu O. , Trifid Mandibular Condyle: A Case Report, Oral Surgery, Oral Medicine, Oral Pathology, Oral Radiology, and Endodontics. (2003) 95, no. 2, 251–254, 10.1067/moe.2003.93, 12582368. 12582368

[18] Stadnicki G. , Congenital Double Condyle of the Mandible Causing Temporomandibular Joint Ankylosis: Report of Case, Journal of Oral Surgery. (1971) 29, no. 3, 208–211, 5278872. 5278872

[19] Ertas E. T. , Sahman H. , and Atici M. Y. , Bilateral Bifid Mandibular Condyle: Report of a Case With Condylar Fractures, Journal of Oral and Maxillofacial Radiology. (2013) 1, no. 2, 80–82, 10.4103/2321-3841.120127.

[20] Zoabi N. , Kats L. , Ram A. , and Emodi-Perlman A. , Trifid Mandibular Condyle: Case Report and Current Review of the Literature, Life. (2022) 12, no. 7, 10.3390/life12070976, 35888066. PMC9317996 35888066

[21] Menezes A. V. , de Moraes R. F. M. , de Vasconcelos-Filho J. O. , Kurita L. M. , de Almeida S. M. , and Haiter-Neto F. , The Prevalence of Bifid Mandibular Condyle Detected in a Brazilian Population, Dentomaxillofacial Radiology. (2008) 37, no. 4, 220–223, 10.1259/dmfr/72314113, 18460575. 18460575

[22] Motta-Junior J. , Aita T. G. , Pereira-Stabile C. L. , and Stabile G. A. , Congenital Frey′s Syndrome Associated With Nontraumatic Bilateral Trifid Mandibular Condyle, International Journal of Oral and Maxillofacial Surgery. (2013) 42, no. 2, 237–239, 10.1016/j.ijom.2012.06.016, 22832663. 22832663

[23] Sampaio-Neves F. , Ramírez-Sotelo L. R. , Roque-Torres G. , Barbosa G. L. R. , Haiter-Neto F. , and de Freitas D. Q. , Detection of Bifid Mandibular Condyle by Panoramic Radiography and Cone Beam Computed Tomography, Brazilian Journal of Oral Sciences. (2013) 12, 16–19.

[24] Khojastepour L. , Kolahi S. , Panahi N. , and Haghnegahdar A. , Cone Beam Computed Tomographic Assessment of Bifid Mandibular Condyle, Journal of Dentistry. (2015) 12, no. 12, 868–873. 27559345 PMC4983301

[25] Gulati A. , Virmani V. , Ramanathan S. , Verma L. , and Khandelwal N. , Bifid mandibular condyle with Temporomandibular Joint ankylosis: Report of Two Cases and review of Literature, Skeletal Radiology. (2009) 38, no. 10, 1023–1025, 10.1007/s00256-009-0724-8, 19521698. 19521698

